# Caddyshack in the Illawarra Shoalhaven: Evaluating a Multi‐Platform Sexual Health Promotion Programme

**DOI:** 10.1002/hpja.70256

**Published:** 2026-10-20

**Authors:** Daniel Demant, Maddison Stratten, Winifred Adamson, Nikki Percival

**Affiliations:** ^1^ School of Human Performance, Rehabilitation and Population Health, Faculty of Health University of Technology Sydney Ultimo Australia; ^2^ School of Public Health and Social Work, Faculty of Health Queensland University of Technology Brisbane Australia; ^3^ Illawarra Shoalhaven Local Health District HIV & Related Programs Unit (HARP) Wollongong New South Wales Australia

**Keywords:** adolescent health services, health promotion, reproductive health, sexual health, social media

## Abstract

**Issue:**

Young people in Australia experience substantial barriers to sexual and reproductive health (SRH) information and services, contributing to high rates of sexually transmissible infections (STIs). Caddyshack Project, developed by the HIV and Related Programs (HARP) Unit in the Illawarra Shoalhaven Local Health District (ISLHD), is a multi‐component health promotion programme that combines digital and traditional outreach to enhance knowledge and accessibility of SRH resources.

**Methods:**

This mixed‐methods evaluation included an online survey with parents, end‐users and multiplicators, focus groups with young people aged 18–24 and social media analytics across Facebook and Instagram.

**Results:**

Sixty‐nine people responded to the survey, the majority being multiplicators such as teachers and health workers. Awareness of programme components varied, with newsletters and webinars most commonly known, while the highest usage was for ordering sexual health products. Satisfaction was consistently high, particularly, for educational materials and practical resources. Focus group participants valued bold designs and practical items but expressed concerns about inclusivity and branding. Social media analysis indicated Instagram reached a broader audience, whereas Facebook resonated more with professionals and parents.

**Conclusions:**

Caddyshack was perceived as trusted and relevant by participants that combine community credibility with digital innovation. However, improvements are needed in outreach, inclusivity and branding to maximise impact.

**What This Study Adds:**

These findings highlight the importance of co‐designed, multi‐platform health promotion strategies that balance professional credibility with youth‐focused engagement in order to address ongoing inequities in SRH access.

## 1 Introduction

Sexual and reproductive health (SRH) remains a significant public health issue in Australia, with bacterial sexually transmissible infections (STIs), including chlamydia, gonorrhoea and syphilis, continuing to increase, particularly, among young people [1, 2]. Young people often face barriers to SRH information and care, including stigma, limited health literacy and structural inequities, with these challenges particularly affecting LGBTQ+ populations and culturally and linguistically diverse communities [3].

In New South Wales, policy frameworks such as the NSW HIV Strategy 2021–2025 and NSW STI Strategy 2022–2026 have prioritised prevention, stigma reduction, youth engagement and digital innovation [4, 5]. Digital and peer‐based approaches are now widely used in efforts to engage young people in SRH promotion, with digital interventions offering opportunities to expand reach and improve sexual health knowledge, although evidence for effects on behavioural intentions is limited [6].

The Caddyshack Project was developed within this context by the HIV and Related Programs (HARP) Unit in the Illawarra Shoalhaven Local Health District (ISLHD) as a youth‐oriented SRH promotion programme combining digital engagement, educational resources and community outreach. This evaluation aimed to examine the reach, perceived effectiveness and relevance of the project for young people and key community stakeholders.

## 2 Programme Overview

Caddyshack was established in 2007 as a sexual health promotion programme to provide young people with knowledge, tools and resources to support safe and healthy sexual practices. The programme also seeks to equip parents, teachers, health professionals and community workers with resources that enable them to act as multiplicators, that is, intermediaries who extend the programme's reach through their professional or community roles.

The programme combines several components reaching end‐users through multiplicators, including 2415 monthly newsletter and blog subscribers, 1415 On The Couch webinar participants and 41 210 vodcast/podcast listeners, alongside educational resources, branded merchandise and sexual health products such as condoms and lubricants distributed through 287 ISLHD services and organisations until September 2026. On The Couch is a digital series that hosts guests to discuss current social themes and sex‐positive topics. Digital engagement is central to the programme, with active social media accounts including Facebook and Instagram reaching 761 and 986 followers respectively to date and the development of online educational and referral resources such as the Youth Empowerment Tool. These are complemented by in‐person outreach activities such as community events, competitions and small group education sessions. This multi‐layered approach aims to normalise discussions of sexual health, improve knowledge, provide referral pathways to local and state‐wide services and reduce sexuality‐associated stigma.

## 3 Methods

A mixed‐methods evaluation was conducted in 2023/2024 to examine the reach, perceived effectiveness and relevance of the Caddyshack project and its activities and resources. The evaluation combined an anonymous online survey, qualitative focus groups and a social media analysis to capture breadth and depth of perspectives on the programme. Ethical approval was obtained from the Greater Western Human Research Ethics Committee (2023ETH02655) and ratified by the University of Technology Sydney Health and Medical Research Ethics Committee.

The survey was distributed online targeting three groups: young end‐users who had used resources themselves, multiplicators who used the programme professionally (e.g., teachers and health/community workers) and parents. Recruitment occurred through email invitations to individuals registered with Caddyshack activities and through promotional posts on social media and newsletters. As recruitment included open invitations, a response rate cannot be calculated. The survey assessed awareness and use of programme components, levels of satisfaction, perceptions of the brand and included open‐ended questions for qualitative feedback.

Two in‐depth focus groups were conducted with young people aged 18–24 years, recruited through social media and educational networks. Each group consisted of four to six participants and explored awareness, first impressions, design elements, inclusivity and usability of Caddyshack's resources. No additional demographic characteristics were collected. Discussions were transcribed and analysed thematically to identify recurring patterns and insights, following Braun and Clarke's approach [7]. No specific research software was used to analyse qualitative data. The focus groups were exploratory in nature and thematic saturation was therefore not used to determine sample size.

Social media performance was assessed using Facebook and Instagram Insights and routine social media data provided by the ISLHD through a manual analysis of 258 unique posts (Facebook: *n* = 142; Instagram: *n* = 116) over a 12‐month period (August 2023 to July 2024). Posts were categorised by content type and engagement scores were calculated using likes, shares and comments. This allowed for a comparison of platform performance and identification of audience engagement trends.

## 4 Findings

### 4.1 Survey

A total of 69 people completed the survey (see Table 1). The majority were multiplicators; accordingly, survey findings predominantly reflect the perspectives of professionals and community stakeholders engaging with Caddyshack rather than young end‐users. Multiplicators primarily worked in health services (49%) and community organisations (30%), with smaller proportions working in educational settings.

**TABLE 1:** Characteristics of participants.

Participant characteristic	*n* (%)^a^
Respondent role (*N* = 69)	
Multiplicator	58 (84.1)
End‐user	8 (11.6)
Parent	3 (4.3)
Multiplicators	
Work environment	
Education/school	8 (15)
Health services	26 (49)
Community (health) organisation	16 (30)
Other	3 (6)
Education/school	8 (15)
Discussed Caddyshack with colleagues	36 (71)
Became aware of Caddyshack through	
Work (e.g., newsletter)	23 (39)
Colleague	20 (34)
Social media	5 (9)
Professional society or interest group	4 (7)
Other	7 (11)
End‐users	
Discussed Caddyshack with friends	3 (50)
End‐users and parents	
Became aware of Caddyshack through	
Social media	5 (63)
All other sources	3 (37)

^a^ Valid responses/percentages.

Awareness of programme components varied (see Table 2). The monthly newsletter was the most widely recognised (67%), followed by webinars (59%) and merchandise (58%). Social media presence was recognised by 52% of respondents, while the Youth Empowerment Tool was least known (28%). Usage among those aware of each resource was generally high. For instance, 91% of respondents aware of the option to order sexual health products had used it.

**TABLE 2:** Awareness and Use of caddyshack components.

Component	Awareness (*n* = 69)	Used		Satisfaction^b^		
		All	Those aware^a^	Exceeded expectations	Met expectations	Below/failed expectations
On‐the‐couch webinars and recordings	59% (*n* = 41)	45% (*n* = 31)	76%	40% (*n* = 12)	47% (*n* = 14)	13% (*n* = 4)
Ordering merch	58% (*n* = 40)	46.4% (*n* = 32)	80%	50% (*n* = 16)	25% (*n* = 8)	25% (*n* = 8)
Ordering sexual health products	48% (n 33)	43.5% (*n* = 30)	91%	66% (*n* = 19)	24% (*n* = 7)	20% (*n* = 3)
Ordering educational materials	44% (*n* = 30)	30.4% (*n* = 21)	70%	71% (*n* = 12)	n/a	29% (*n* = 5)
Monthly newsletter	67% (*n* = 46)	54% (*n* = 37)	80%	32% (*n* = 12)	57% (*n* = 21)	11% (*n* = 4)
Monthly blog	42% (*n* = 29)	26.1% (*n* = 18)	62%	22% (*n* = 4)	78% (*n* = 14)	n/a
Social media presence	52% (*n* = 36)	34.8% (*n* = 24)	67%	35% (*n* = 8)	57% (*n* = 13)	8% (*n* = 2)
Youth empowerment tool	28% (*n* = 19)	10.1% (*n* = 8)	37%	62% (*n* = 5)	38% (*n* = 3)	n/a

^a^ Based on valid responses to awareness of component existing.

^b^ Based on valid responses to the satisfaction item for each component; denominators therefore vary by component.

Satisfaction with the quality of Caddyshack's resources was strong. Educational materials received the highest ratings, with 71% of respondents reporting that they exceeded expectations. Most other components, including sexual health product orders (66% exceeding expectations) and webinars (40% exceeding expectations), met or exceeded expectations, with relatively few respondents indicating disappointment.

Respondents' perceptions of Caddyshack as a brand were generally positive. The brand was rated highly for being trustworthy, with a mean agreement score of 4.5 (SD = 0.8) on a scale from 1 (*strongly disagree*) to 5 (*strongly agree*). It was also seen as modern (mean = 4.3, SD = 0.9), innovative (mean = 4.2, SD = 0.9) and relevant (mean = 4.2, SD = 1.0).

Survey comments emphasised three recurring themes. First, the need for greater inclusivity and diversity was highlighted, particularly in relation to culturally and linguistically diverse populations and refugee communities. Respondents suggested that resources in multiple languages and more discreet options for conservative communities would broaden accessibility. Second, respondents requested structured lesson plans and activity guides to support the use of materials in schools and community settings. Third, respondents encouraged more public engagement activities, including face‐to‐face events and improved promotion of existing tools.

### 4.2 Focus Groups

Focus group participants had limited prior awareness of Caddyshack, which is not unexpected given the large number of health promotion programmes competing for attention and the regional focus of Caddyshack. Despite this, respondents responded positively to the visual appeal, accessibility and practical orientation of programme materials. The bold use of colour and practical items such as lip balm and condoms was received well, although the prominence of pink was interpreted by some as potentially gender‐exclusive, suggesting that branding choices may be interpreted differently by different groups of young people. Participants also expressed a desire for greater diversity in representation across materials and messaging. Instagram was viewed as accessible and engaging, though participants strongly advocated for greater use of TikTok and other contemporary youth‐oriented communication strategies to increase reach and relevance among younger audiences. A recurring theme was the importance of directly involving young people in the co‐design and development of resources to strengthen authenticity, relatability and sustained engagement with target populations.

### 4.3 Social Media Analysis

Between August 2023 and July 2024, Caddyshack's Facebook and Instagram accounts received 6309 post impressions, 3808 post engagements with an engagement rate of 7.37%. During this study period, Instagram followers increased from 655 to 774 (18.2% growth rate), while Facebook followers increased from 745 to 775 (4% growth rate).

Analysis of engagement revealed clear differences between Facebook and Instagram. On Facebook, community‐oriented and celebratory posts generated the highest levels of engagement, whereas informational posts on STI prevention performed less well. For example, high‐performing community and professional posts achieved engagement scores of 50–53, while STI‐related informational content generated minimal engagement. Engagement on the platform was also concentrated among repeat users, suggesting Facebook primarily functions as a tool for maintaining relationships with existing professional, community and parental audiences rather than expanding reach to new groups.

Instagram, by contrast, reached a broader range of users. Posts incorporating dynamic audio‐visual content consistently outperformed static images, while collaborations with influencers and community organisations generated particularly strong traction and audience interaction. The highest‐engagement Instagram post, a video promoting the Indigenous surfboard competition, achieved an engagement score of 73, compared with 12 for a static informational post. Instagram also attracted a larger proportion of unique users and more diverse audience engagement overall, suggesting it may be more useful for expanding reach beyond existing audiences.

## 5 Discussion

This evaluation found that Caddyshack was generally well regarded by participants and that its combination of practical resources, digital engagement and community outreach was viewed positively. Participants consistently described the programme as trustworthy, relevant and accessible, while educational materials and sexual health product distribution were particularly highly valued. The findings indicate that participants valued both the practical support offered through the programme and the information it provided, aligning closely with the prevention, stigma reduction and youth engagement priorities outlined in the NSW HIV and STI strategies [4, 5].

However, the evaluation also identified several challenges relating to visibility, inclusivity and audience engagement. Awareness of Caddyshack among young end‐users remained comparatively limited, despite strong perceptions among those familiar with the programme. This suggests that while the programme is well‐regarded by existing audiences, expanding direct reach to young people remains a key challenge, consistent with findings in the broader sector [8]. However, it should be noted that multiplicators are also an intended audience, as they share Caddyshack resources with young people; however, because they comprised 84% of survey respondents, the survey findings primarily reflect their perspectives rather than those of young end‐users. The findings from the social media analysis reinforce this with Facebook primarily functioning as a platform for maintaining engagement with established professional, community and parental audiences, whereas Instagram demonstrated greater capacity to reach new and more diverse audiences, particularly through dynamic audio‐visual content and collaborations with community organisations and influencers. These findings show the importance of platform‐specific engagement strategies rather than uniform approaches across social media environments [9].

The results also highlight the importance of co‐design and inclusive communication strategies in contemporary sexual health promotion, including approaches that reflect young people's preferences and the cultural and linguistic diversity of their intended audiences [10, 11]. Participants valued the bold and youth‐oriented branding of Caddyshack, yet some design choices, particularly the perceived prominence of pink and limited diversity in representation, were interpreted as potentially exclusionary. Similarly, participants identified the absence of multilingual and more discreet resources for culturally and linguistically diverse and refugee communities as a limitation. Without inclusive development, some groups may continue to face barriers to sexual health information. Direct involvement of young people in co‐design processes was repeatedly identified as essential for ensuring authenticity, relatability and relevance.

Overall, the evaluation suggests that Caddyshack provides an established platform for youth‐focused sexual health promotion, with positive perceptions among those who engage with the programme but limited broader awareness among young people. However, several practical implications emerge from the findings. First, strengthening direct engagement with young people should remain a priority, including through partnerships with schools, universities, youth organisations and community groups. Second, the development of structured lesson plans and implementation resources may strengthen uptake among educators and health professionals acting as multiplicators. Third, culturally inclusive approaches, including multilingual materials and tailored engagement strategies, are necessary to improve accessibility and reduce inequities in sexual health information and care. Finally, the findings highlight the need for adaptive digital strategies that respond to changing youth media consumption patterns. While Instagram currently presents a promising engagement platform, participants strongly advocated for greater use of emerging platforms such as TikTok and for increased use of dynamic audio‐visual content.

## 6 Limitations

Several limitations should be considered. The small survey sample, predominantly comprising multiplicators and recruitment through existing programme networks may have introduced selection bias towards those already familiar with Caddyshack. Detailed demographic data were not collected, limiting characterisation of survey respondents. The small focus‐group sample may limit generalisability. Finally, the evaluation assessed reach, engagement and perceived value rather than behavioural, knowledge, or clinical outcomes.

## Funding

This work was funded through the Illawarra Shoalhaven Local Health District.

## Ethics Statement

Ethical approval for the study was obtained from the Greater Western Human Research Ethics Committee (Reference: 2023/STE04259), with ratification from the University of Technology Sydney Medical Research Ethics Committee. Data collection and analysis adhered to strict confidentiality and data security protocols to ensure participant privacy and compliance with ethical standards. A waiver of consent has been granted for the analysis of social media data.

## Conflicts of Interest

The Illawarra Shoalhaven Local Health District (ISLHD) has ownership of the Caddyshack programme and was engaged as a research partner, contributing contextual information about the programme, supporting participant recruitment and providing feedback on aspects of the study. While ISLHD was actively involved in supporting the research process, all aspects of study design, data collection, analysis and interpretation were conducted independently by the research team (D.D. and N.P.). Two of the co‐authors, M.S. and W.A., are employed by ISLHD. They did not attend the focus groups, nor did they analyse or have access to the focus group transcripts. Their role was limited to providing contextual information throughout the research process and assisting with the preparation of the manuscript, but they were not involved in the interpretation of the data.

Each author certifies that their contribution to this work meets the standards of the International Committee of Medical Journal Editors.

## Data Availability

The data that support the findings of this study are available on request from the corresponding author. The data are not publicly available due to privacy or ethical restrictions.
